# Insights into the *Planktothrix* genus: Genomic and metabolic comparison of benthic and planktic strains

**DOI:** 10.1038/srep41181

**Published:** 2017-01-24

**Authors:** Claire Pancrace, Marie-Anne Barny, Reiko Ueoka, Alexandra Calteau, Thibault Scalvenzi, Jacques Pédron, Valérie Barbe, Joern Piel, Jean-François Humbert, Muriel Gugger

**Affiliations:** 1 Institut Pasteur, Collection des Cyanobactéries, 28 rue du Dr Roux, 75724 Paris Cedex 05, France; 2 UMR UPMC 113, CNRS 7618, IRD 242, INRA 1392, PARIS 7 113, UPEC, IEES Paris, 4 Place Jussieu, 75005, Paris, France; 3 Université Pierre et Marie Curie (UPMC), 4 Place Jussieu, 75005, Paris, France; 4 Institute of Microbiology, Eigenössische Technische Hochschule (ETH) Zurich, Vladimir-Prelog-Weg 4, 8093 Zurich, Switzerland; 5 Commissariat à l’Energie Atomique et aux Energies Alternatives (CEA), Genoscope & CNRS, UMR 8030, Laboratoire d’Analyse Bioinformatique en Génomique et Métabolisme, 2, rue Gaston Crémieux, CP 5706, 91057 EVRY cedex, France; 6 Commissariat à l’Energie Atomique et aux Energies Alternatives (CEA), Genoscope, Laboratoire de Biologie Moléculaire pour l’étude des Génomes, 2, rue Gaston Crémieux, CP 5706, 91057 EVRY cedex, France

## Abstract

*Planktothrix* is a dominant cyanobacterial genus forming toxic blooms in temperate freshwater ecosystems. We sequenced the genome of planktic and non planktic *Planktothrix* strains to better represent this genus diversity and life style at the genomic level. Benthic and biphasic strains are rooting the *Planktothrix* phylogenetic tree and widely expand the pangenome of this genus. We further investigated *in silico* the genetic potential dedicated to gas vesicles production, nitrogen fixation as well as natural product synthesis and conducted complementary experimental tests by cell culture, microscopy and mass spectrometry. Significant differences for the investigated features could be evidenced between strains of different life styles. The benthic *Planktothrix* strains showed unexpected characteristics such as buoyancy, nitrogen fixation capacity and unique natural product features. In comparison with *Microcystis*, another dominant toxic bloom-forming genus in freshwater ecosystem, different evolutionary strategies were highlighted notably as *Planktothrix* exhibits an overall greater genetic diversity but a smaller genomic plasticity than *Microcystis*. Our results are shedding light on *Planktothrix* evolution, phylogeny and physiology in the frame of their diverse life styles.

Cyanobacteria is a fascinating phylum considering the large diversity of morphological, physiological, ecological, toxicological and genetic characteristics of these microorganisms (e.g. Bothe *et al*.^1^, Calteau *et al*.^2^, Careya *et al*.^3^). This diversity has motivated a huge number of publications in the last decades but many questions of great interest concerning for example the development of multicellularity^4^ or the diversification of life styles into this phylum^5^ are still far from being resolved. Similarly, the taxonomy of Cyanobacteria remains uncertain for many groups.

In recent years, numerous genomes from diverse cyanobacterial genera permitted to enlarge our vision on the genomic diversity of cyanobacteria^6^. This concerned in particular filamentous cyanobacteria, which were poorly investigated until now. Classified in the order Oscillatoriales^7^, the filamentous cyanobacteria are indeed widely dispersed in the radiation of the cyanobacterial phylum^6^, and they display various physiological capacities (for example, diazotrophy, motility and salt tolerance) and ecological strategies for niche adaptation.

Within Oscillatoriales, the genus *Planktothrix* was formed to contain free-floating solitary trichomes distinguished in nine planktic species^8^. However, a benthic strain was recently isolated in a freshwater biofilm in New Zealand^9^, further completed by a larger set of benthic *Planktothrix* strains closely related to the planktic forms of *Planktothrix*^10^. As observed for the heterocystous genus *Nodularia*^11^, the coexistence of planktic and benthic species in the genus *Planktothrix* makes it a good candidate for studying genome adaptation linked to these two ways of life.

The genus *Planktothrix* is also well investigated because it blooms frequently in freshwater ecosystems of temperate areas. This motivated a lot of works on the buoyant capabilities^12^,^13^,^14^,^15^ of the planktic isolates considering their vertical migration in the water column. One of the recently described benthic species was named with respect to its gas vesicle characteristics (*Planktothrix paucivesiculata* PCC 9631)^10^, raising questions on the relevance of gas vesicles in a benthic way of life. To date, no information is available on the genetic basis of buoyancy through the whole genus including the biphasic and benthic strains.

The recurrent toxicity of *Planktothrix* blooms drove most of the works to be performed on their natural products potential, in particular on the biosynthesis of various microcystin variants (e.g. Briand *et al*.^16^, Kurmayer & Gumpenberger^17^). Beyond microcystins, *Planktothrix* is also able to produce others natural products through versatile non-ribosomal peptide synthetase (NRPS) and polyketide synthase (PKS) biosynthetic pathways or as ribosomally synthesized and post-translationally modified peptides (RiPPs)^18^,^19^,^20^,^21^. Notably, a compound previously unrelated to this genus has been recently found in *Planktothrix paucivesiculata* PCC 9631^21^ leading us to expect more unusual *Planktothrix* compounds in their various life styles.

This study aims (i) at better representing the *Planktothrix* genus diversity and life style at the genomic level and (ii) at investigating the genetic basis behind few common and divergent metabolic features between benthic and planktic strains. In this frame and in complement to the recent genomic data obtained for nine closely related planktic *Planktothrix* forming blooms in Nordic lakes^22^,^23^, we sequenced the genomes of six strains from the Pasteur Culture Collection of Cyanobacteria (PCC) including four biphasic/benthic strains. We investigated the capability of these strains to produce aerotopes and their capacity to fix nitrogen. We also examined their genetic potential for natural products to find the diverse pathways present in this oscillatorialean genus.

## Results

### General features of the six *Planktothrix* genomes studied

The general features of the six *Planktothrix* strains, their appearances in cultures and their genomes sequenced in this study are presented in Table 1, Fig. S1 and Table 2. The complete *P. agardhii* PCC 7805 genome contains one chromosome (4.7 Mb), one megaplasmid (151 kb) and one cryptic plasmid (4.5 kb). The simultaneous presence of a resolvase gene (*rsvA*; PLAM_mp0003) and a homolog to *tnpA* transposase (PLAM_mp0004) associated to a fold-coverage comparable to that of the chromosome, suggested that the megaplasmid could be integrative. Using PCC 7805 genome as reference, we identified the scaffolds belonging to the chromosome, the megaplasmid and the plasmid in *P. rubescens* PCC 7821 (Table 2). However, the resolvase and *tnpA*-like genes were not found into PCC 7821 megaplasmid, although fold-coverage was the same than that of the chromosome. The genome size of the benthic/biphasic life style strains, between 5.9 Mb (*Planktothrix* sp. PCC 11201) and 6.7 Mb (*P. tepida* PCC 9214), was larger than the average genome size of planktic strains (Table 2).

Our *Planktothrix* genomes were compared to the nine *Planktothrix* genomes from Nordic lakes already available. Using the genome of PCC 7805 as reference, the genomes of the planktic strains showed synteny values above 80%, whereas the ones of the four *Planktothrix* with another life style were slightly lower (Fig. S2). A non-metric multidimensional scaling (NMDS) analysis on the clusters of orthologous groups (COG) further revealed that most of the Nordic *Planktothrix* grouped together whereas two planktic *Planktothrix* PCC 7805 and NIVA CYA 126/8, two *Planktothrix* PCC 9214 and PCC 8927 and the two *Planktothrix* PCC 11201 and PCC 9631 formed three distinguished groups apart (Fig. S3). The overall comparison of the COG showed an enrichment of the benthic and biphasic strains in genes related to signal transduction mechanisms (Table S1). In addition, 38 to 48 tRNAs were found in the *Planktothrix* genomes, the benthic and biphasic strains having more tRNA than the planktic ones (Table S2).

**Figure 1:**
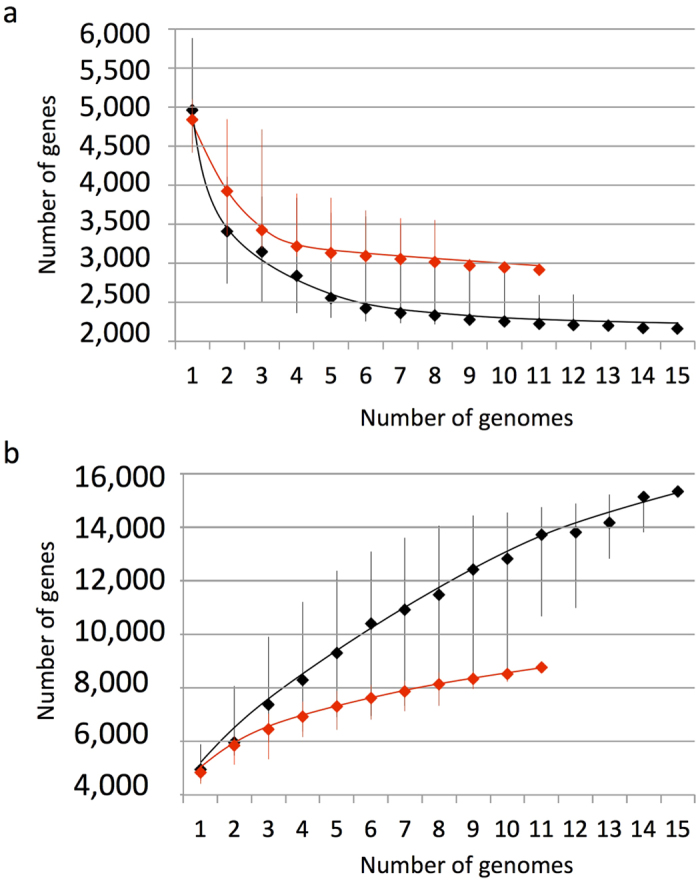
Sizes estimation of the core genome in number of conserved genes (**a**) and pangenome in number of specific genes (**b**) of all *Planktothrix* (in black) and the planktic strains only (in red).

The core and pan genome analysis of all *Planktothrix* genomes revealed about 2,200 genes for the core, and about 3,000 genes when considering only the planktic ones (Fig. 1a). With regard to the pangenome, the gene accumulation curve did not reach a plateau when considering all genomes, while the almost asymptotic curve obtained with genomes from planktic strains suggested between 8,000 and 9,000 genes in their pangenome (Fig. 1b).

**Figure 2:**
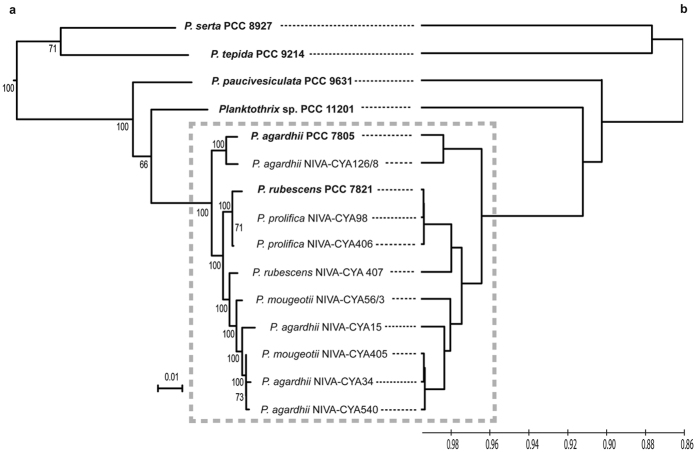
Genetic relationships between the fifteen available *Planktothrix* genomes. (**a**) Phylogenetic tree obtained using a Maximum Likelihood method on 586 concatenated proteins.(**b**) Phenetic tree (UPGMA) obtained from the ANIm values between all genomes. Grey rectangle contains all the planktic strains.

Finally, the genetic relationship between our six strains and the nine available planktic *Planktothrix* was studied by a Maximum-Likelihood phylogenetic approach performed on 586 concatenated proteins from the core genomes and by a phenetic approach (UPGMA) performed on the average nucleotide identity values. A good overall congruence between the trees was observed with a well-defined cluster containing all the planktic strains rooted by the four non planktic strains at the basis of the tree (Fig. 2). An extended phylogeny on 29 genes with the closest cyanobacterial relatives indicated the same topology further rooted by a cluster with three *Arthrospira* and the marine *Lyngbya* sp. CCY 9616 (Fig. S4). The same tree topology was obtained using the 16 S rRNA gene sequences (data not shown). From these findings, it appeared that the eleven planktic strains (nine previously sequenced and two new ones) belong to the same species while the four benthic or biphasic strains at the tip of longer branches are more heterogeneous and belong to different species.

### Gas vesicles in planktic and benthic forms of *Planktothrix*

**Figure 3:**
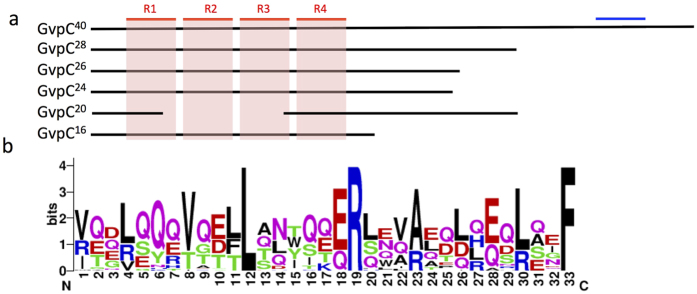
Scheme of the structure of GvpC proteins of *Planktothrix*. (**a**) Alignment of GvpC protein variants of *Planktothrix* sp. with GvpC^40^ from PCC 11201, PCC 8927, PCC 9214 and PCC 9631, GvpC^28^ from *P. agardhii* PCC 7805 and *P. mougeotti* NIVA CYA 56-3, GvpC^26^ from NIVA CYA 34 and NIVA CYA 405, GvpC^24^ from NIVA CYA 15, GvpC^20^ from PCC 7821 and GvpC^16^ from NIVA CYA 540. The red lines indicate the localisation of the four degenerated repeats R1, R2, R3, and R4. The blue line indicate the N terminal domain presenting homology with GvpN; (**b**) Consensus sequence found between the R1, R2, R3, and R4 repeats. LRF residues are conserved in *Anabaena* and *Calothrix* repeats as well^49^.

The gas vesicles are essential for the floatability of planktic *Planktothrix*, but whether they are needed in the life style of the benthic *Planktothrix* remained to be investigated. The *gvp* gene clusters were present in all *Planktothrix* genomes whether the strains had a planktic, benthic or biphasic life style. The quality of the genome assemblies and the different sequencing techniques did not allow analyzing the organization of the *gvp* gene clusters. However, the genes *gvpA, gvpC, gvpN, gvpJ, gvpK, gvpF*/*L, gvpG* and *gvpV* were found in all *Planktothrix* genomes, while the gene *gvpW* was absent only from the genome of benthic strain PCC 11201. The GvpC protein being a structurant element of the size of the gas vesicles, we examined the diversity of the *gvpC* gene of our *Planktothrix* dataset. Indeed, the *gvpC* gene appeared to code more GvpC protein variants than expected. The three commonly described length variants of 16 kDa GvpC in *P. prolifica* NIVA CYA 540, 20.1 kDa GvpC in *P. rubescens* PCC 7821 and 28.6 kDa GvpC in *P. mougeotii* NIVA CYA 56-3 and *P. agardhii* PCC 7805 were completed with a 24.3 kDa GvpC in *P. agardhii* NIVA CYA 15, a 26.3 kDa GvpC in *P. mougeotii* NIVA CYA 405 and in *P. agardhii* NIVA CYA 34, and a larger GvpC variant of about 40 kDa in all non planktic strains (39.8 kDa GvpC in *Planktothrix sp*. PCC 11201 and 39.5 kDa GvpC in *P. serta* PCC 8927, *P. tepida* PCC 9214 and *P. paucivesiculata* PCC 9631). The phylogeny of the GvpC protein sequences of *Planktothrix* (Fig. S5) is congruent with the one obtained for the taxonomic markers, indicating that GvpC is likely vertically inherited. Aware of the GvpC size discrepancy between planktic and non planktic *Planktothrix*, we investigated the gas vesicles of the benthic *Planktothrix* sp. PCC 11201 and the planktic *P. agardhii* PCC 7805, which did not shown any difference in abundance and size of their aerotopes (Fig. S6). Interestingly, the alignment of GvpC^40^ with the smallest GvpC proteins revealed 33 amino acid long degenerated motifs repeated (Fig. 3a). These GvpC motifs present in four repeats in GvpC^16,24,26,28,40^ contained invariant amino acids at positions 12, 19 and 33 similar to the ones described for the repeated motifs of *Anabaena* and *Calothrix* GvpC (Fig. 3b). The motif was also present in the N-terminal part of truncated GvpC^20^ of PCC 7821, but it would not have been found without comparison with the other *Planktothrix* GvpC variants. In addition, the C-terminal GvpC^40^ was similar at 78% to a 60 amino acid long sequence of the C-terminal GvpC of 40.5 kDa in *Geitlerinema* sp. PCC 7407 (WP_015171019.1) and, a 28 amino acid stretch in the C-terminal GvpC^40^ shared 64% sequence identity with the protein GvpN of *Planktothrix* (Fig. 3a).

### Nitrogen fixation in *Planktothrix* strains

**Figure 4:**
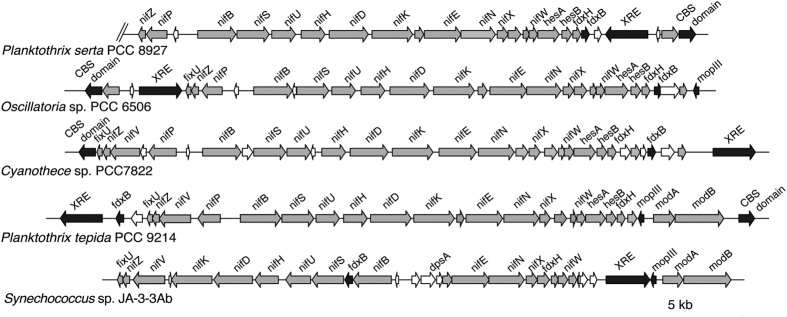
Organization of the *Nif* gene clusters in two benthic *Planktothrix* strains PCC 8927 and PCC 9214 compared to the ones in other non-heterocystous cyanobacteria. The conserved genes of the *nif* cluster locus are indicated in grey, the genes indicated in black are present and rearranged between the clusters, the genes indicated in white are not conserved.

Among all the *Planktothrix* genomes studied, *P. tepida* PCC 9214 and *P. serta* PCC 8927 harboured the entire *nif* gene cluster (Fig. 4). These clusters contained the nitrogenase structural genes *nifH, nifD*, and *nifK* flanked on the left by the *nifU, nifS, nifB, nifP* and *nifZ* genes and on the right by *nifE, nifN, nifX, nifW, hesA, hesB*, and *fdxH* genes. This general organization of the *nif* gene cluster was identical in *Cyanothece* sp. PCC 7822 and *Oscillatoria* sp. PCC 6506, but differed from the one of *Synechococcus* sp. JA-3-3Ab. The genes *nifV* and *fixU* were not included in the *nif* cluster of *P. serta* PCC 8927 but found elsewhere in the genome. Interestingly, both *P. serta* PCC 8927 and *P. tepida* PCC 9214 *nif* clusters were flanked by the *fdxB* gene and a gene encoding a XRE (Xenobiotique response element) family transcriptional regulator. This regulator, named *PatB* in heterocystous cyanobacteria and *CnfR* in other diazotrophic cyanobacteria, was identified as the master regulator for the *nif* genes^24^. Moreover, three molybdenum transporter genes (*modIII, modA*, and *modB*) also flanked the *nif* gene cluster of *P. tepida* PCC 9214, as the one of *Synechococcus* sp. JA-3-3Ab.

A phylogenetic analysis performed on the concatenated sequences of *nifBDHSU* genes of diverse diazotrophic cyanobacteria revealed that the *nif* cluster of PCC 8927 is not closely related to the one of PCC 9214 (Fig. S7). The *nifBDHSU* of *P. serta* PCC 8927 is located in a clade containing the benthic filamentous *Oscillatoria* spp. PCC 6506 and PCC 6407, *Leptolyngbya boryana* PCC 6306, the unicellular *Cyanothece* sp. PCC 7425 and diverse heterocystous strains such as *Anabeana variabilis* ATCC 29413, *Fischerella* spp. PCC 9605 and JSC-11, and *Nodularia spumigena* CCY 9414. On the other hand, the *nifBDHSU* of *P. tepida* PCC 9214 is placed between the clade of the *Synechococcus* from Yellowstone and the clade containing *P. serta* PCC 8927 and other cyanobacterial diazotrophs, and the nodes connecting this strain with these two clades are not supported by bootstrap values. Furthermore, single *nif* gene phylogenies (data not shown) were incongruent with the location PCC 9214 in the *nifBDHSU* tree, while the locus containing *nifBDHSU* genes is conserved in the 26 *nif* gene clusters examined (Fig. S7). This indicated that PCC 9214 hold a mosaic cyanobacterial *nif* cluster. To assess if the *nif* gene clusters were functional in both strains (PCC 9214 and PCC 8927), growth tests were performed under different nitrogen concentrations, with the other *Planktothrix* strains as negative controls and the nitrogen fixer *Cyanothece* sp. PCC 7822 as positive control. All the strains cultivated under continuous light died under nitrogen starvation conditions. When a day/night rhythm was applied, *P. serta* PCC 8927 was able to grow and develop a thick biofilm under nitrogen starvation conditions, but not *P. tepida* PCC 9214 (Fig. S8). This suggested that in our culture conditions, only *P. serta* PCC 8927 was able to fix N_2_.

### Genetic potential dedicated to natural products in the toxic *Planktothrix*

**Figure 5:**
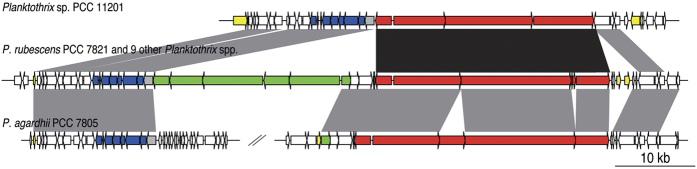
Genomic island dedicated to natural products in *Planktothrix*. Similar genomic islands containing biosynthetic gene clusters of *apt* (green), *oci* (red) and *mdn* (blue) were found in *P*. sp. PCC 11201, *P. rubescens* PCC 7821 identical to the one found in nine planktonic *Planktothrix* spp. (NIVA-CYA 15, NIVA-CYA 34, NIVA-CYA 56/3, NIVA-CYA 98, NIVA-CYA 126/8, NIVA-CYA 405, NIVA-CYA 406, NIVA-CYA 407 and NIVA-CYA 540) and in *P. agardhii* PCC 7805. In the latter, the genomic island is not evidenced by the current assembly of the contigs, but one remaining gene of *apt* and the flanking regions witness a previous occurrence of the same genomic island organization. Clusters with conserved sequence and domain organization are connected in grey and in black when reorganized. The genetic island in addition of *apt, oci* and *mdn* gene clusters contained other genes putatively involved in these pathways (in grey), flanking genes (in white) and genes of mobility such as insertion sequences and transposases (in yellow).

Twenty biosynthetic gene clusters dedicated to natural products (BGCs) were identified in *Planktothrix* genomes (Table 3). The BGCs of 3.4 to 92.8 kb-long belong to RiPPs, NRPS, PKS and hybrids of the two last categories. The strains contained two to nine BGCs accounting for 0.9 to 3.7% of the total genome size. When looking at the distribution of all these BGCs among all the available *Planktothrix* genomes, it appeared that the planktic strains and the benthic strain PCC 11201 grouped together, while the three other benthic strains were much more dispersed revealing drastic differences in their BGC content (Fig. S9). More in details, ten BGCs previously described in *Planktothrix* encoding the biosynthesis of aeruginosin (*aer*), cyanobactin (*pat*), cyanopeptolin (*oci*), luminaolide B (*lum*), microcystin (*mcy*), microviridin (*mdn*), microginin (*mic*), anabaenopeptin (*apt*) oscillatorin (*osc*) and prenylagaramide (*pag*), are conserved with a high amino acid identity (AAI) in this clade (Table 3). The BGCs *apt, mic*, and *oci* appeared localised in a ~66 kb genomic island for several *Planktothrix* strains as they were previously showed co-localised in *Planktothrix rubescens* NIVA CYA 98^19^ (Fig. 5). This genomic island was found in nine other planktic strains (PCC 7821, NIVA-CYA 15, NIVA-CYA 34, NIVA-CYA 56/3, NIVA-CYA 405, NIVA-CYA 406, NIVA-CYA 407, NIVA-CYA 540 and NIVA-CYA 126/8), and more strikingly in the genome of the benthic *Planktothrix* strain PCC 11201. This locus presented the features of a genomic island such as a size >20 kb, the presence of mobile elements (insertion sequence and transposases at their extremities) and a compositional bias (2 mer to 8 mer compositional bias of distribution, codon usage bias, and for PCC 11201 an additional GC percentage deviation (−1 Standard Deviation)). In addition, this genomic island region was split in two regions in *P. agardhii* PCC 7805 with the deletion of most *apt* genes compared to the ten other planktic *Planktothrix*. However, the remaining *apt* gene and the flanking regions witness a previous occurrence of such co-localised organization in the genome of PCC 7805.

Among the other BGCs, two *trans-*AT PKS gene clusters were found in the genomes of PCC 9631 and PCC 11201. The *trans-*AT PKS gene cluster of PCC 9631 encode the biosynthetic pathway for luminaolide B, while the one of PCC 11201 exhibited a homology of 76% to the tolytoxin gene cluster (tto) described for *Scytonema* sp. PCC 10023^21^. We confirmed the production of tolytoxin in PCC 11201 by HPLC, MS, and NMR (Fig. S10). In addition, a *has*-like gene cluster was found in *Planktothrix serta* PCC 8927 with four NRPS genes similar at 66% to the hassallidin gene cluster found in *Anabaena* sp. SYKE748A^25^. However, the characterisation of the other ORFs of this *has*-like gene cluster and the eventual natural product synthesis will need further study.

The other gene clusters displayed variability in their size, gene composition and organization, and precursors (Table 3). Genome mining allowed the comparison of BGCs *pat* and *mic* to homologous clusters in other genomes. The cyanobactin BGC (*pat*-like) of PCC 9631 was similar at 91% to aeruginosamide gene cluster of *Microcystis* sp. PCC 9432, and its structure organisation recalled the ones of microcyclamide cluster of *Microcystis* sp. PCC 7806 and aesturamide cluster of *Lyngbya* sp. CCY 9616^26^,^27^. The structure of these BGCs and comparison of their precursor peptides suggested the production by PCC 9631 of a cyclic cyanobactin with two prenylations (Fig. S11). Similarly, the *mic* BGC on the megaplasmid of PCC 7821 was identical to one encoding microginin in *Planktothrix prolifica* NIVA-CYA 98 and 73% similar to one in *Microcystis aeruginosa* PCC 9432^19^,^28^. The *mic* cluster synteny comparison indicated four additional ORFs coding for a CAL domain, a peptidyl carrier protein domain, an *O*-methyltransferase that might be involved in the synthesis of the microginin (Fig. S12).

The remaining gene clusters were not linked to known natural products. Two orphans PNL1 and PNL2 were previously described in the *Planktothrix* NIVA CYA 98^19^. PNL1 located on the megaplasmid of PCC 7805 shared 96% AAI with *P. prolifica* NIVA-CYA 98 and 66% AAI with *Spirulina major* PCC 6313. Finally, the bacteriocin-like gene cluster RiPP1 was similar to a gene cluster in *Oscillatoria* sp. PCC 10802 (84%). The gene clusters PNL3, PHL1, PPL1, PPL2 and PPL3 had no significant similarity in databases.

## Discussion

Our comparative genomic approach on benthic and planktic *Planktothrix* strains has permitted to clarify the phylogenetic relationships between all these strains. Their ANI values were supporting their placement into the same genus taxa as recently proposed by Gaget *et al*.^10^ by using 16S rRNA and MLST on few other stable genetic markers. Interestingly, this genus appear to be close to *Arthrospira* genus and in a lesser extent to some oscillatorian strains, which contain benthic and planktic species. Inside the *Plantothrix* genus, both ANI values and pan genome data suggest that the 15 planktic strains including green and red ecotypes, belong to a single species, whereas the four benthic and biphasic strains are split into four different species at the root the planktic clade. The diversity observed among the benthic *Planktothrix* recall the one reported for *Nodularia*^11^. These findings are interesting to consider in regard to the data recently obtained showing that the appearance of benthic cyanobacteria precludes the emergency of planktic ones in marine ecosystems^5^. As for marine cyanobacteria, benthic life style might precede planktic life style in freshwater ecosystems, at least for this taxonomical group.

In the *Planktothrix* genome comparison, we paid a special attention to genetic differences between benthic and planktic strains. The genes involved in the biosynthesis of gas vesicles that confer buoyancy to the cells displayed significant variations inside the *Planktothrix* genus. Indeed, all benthic *Planktothrix* shared a GvpC^40^ protein containing four degenerated repeat sequences while planktic strain GvpC were smaller and more diverse in size (from GvpC^16^ to GvpC^28^). The shorter GvpC proteins of the planktic strains kept trace of the four repeats identified in the benthic strains. These variations are interesting in regard to the life style of the strains. As previously shown for planktic strains^12^,^29^, variations in gvpC genes seem to be directly associated to the differentiation of various gas vesicle phenotypes, and consequently, to their adaptation to various environmental conditions. In the benthic strains, the presence of gas vesicles could be useful for the propagation of their filaments or to the detachment of mature biofilms from their support.

Complete *nif* gene clusters were found in two benthic *Planktothrix* strains, but only one exhibited nitrogen fixation capacity in our conditions. The nitrogen fixation is active in several unicellular, filamentous and most of heterocystous cyanobacteria providing advantages over concurrent species, in oligotrophic environments or with the increase of atmospheric CO_2_^30^. Nitrogen fixation was mostly studied in *Trichodesmium* and *Cyanothece* proliferating in nitrogen-depleted ocean, and in *Anabaena* for characterizing the cellular differentiation dedicated to this process^30^,^31^,^32^. However, this capacity was also reported in benthic cyanobacteria of the genus *Hydrocoleum*^33^. In our experimental conditions, only *Planktothrix serta* PCC 8927 was able to fix N_2_ as shown by its ability to grow in a medium depeleted in nitrogen. The other strain containing the complete *nif* gene cluster, *Planktothrix tepida* PCC 9214, was unable to grow in this medium, and may need as several filamentous non heterocystous strains belonging to the genera *Phormidium, Pseudanabaena* and *Oscillatoria*, micro-oxic or anoxic conditions to fix N_2_^34^. As the other non planktic *Planktothrix* studied did not fix nitrogen, this capacity seems not linked to their life style, even if it is known that benthic microorganisms are mostly found in oligotrophic water containing low nitrogen concentrations. As the *Planktothrix* clade is engulfed into a larger oscillatorian clade comprising several diazotrophic cyanobacteria (Fig. S4), the evolutionary origin of the *nif* in *Planktothrix* seems coherent with the vertical inheritance of diazotrophy in most of cyanobacteria, with repeated loss in non nitrogen-fixers^35^, and *nif* gene cluster rearrangements such as in PCC 9214.

Concerning the potential for the production of natural products, significant differences were found between the benthic and planktic strains. Interestingly, the benthic strain PCC 11201, which is phylogenetically more closely related to the planktic strains, shared numerous BGCs with them while the three other non planktic strains displayed more difference in their potential to produce secondary metabolites. In particular, PCC 11201 contains a large portion of the genomic island comprising anabeanopeptin, cyanopeptolin and microviridin that is also present in most of the genomes of the free-living *Planktothrix*. This co-localization was previously reported in the genome of *Planktothrix* NIVA CYA 98^19^, whereas genomic islands in cyanobacteria were previously associated to the transfer of light harvesting phycobilisome rod complexes or to glycosyltransferases and transport proteins^36^,^37^. Such a large genomic island dedicated to two NRPS and one RiPP in *Planktothrix* was not reported previously in the cyanobacterial BGCs studied at phylum-wide level^2^. However, other BGC arrangements on genomic islands were described in other bacteria^27^,^38^. Whether the co-localization is linked to the co-regulation of these BGCs remains to be investigated. Several other BGCs encoded for yet unknown natural products, while several *Planktothrix* compounds are not related to any genetic data. Indeed, the comparisons of unknown BGCs lead to those from highly unrelated organisms such as myxobacteria from soil and *Entotheonella*-symbiont of marin sponges^21^. We uncovered in *Planktothrix* sp. PCC 11201 the polyketide tolytoxin, typically found otherwise in heterocystous cyanobacteria such as *Tolypothrix* and *Scytonema*. Similarly, a close congener of luminaolide B recently described from *Planktothrix paucivesiculata* PCC 9631 was previously reported from a marine coral-consortium^21^. Both tolytoxin and luminaolide B are produced through related *trans*-AT PKSs^21^, an enzyme family that seems to occur more often in the cyanobacterial phylum than previously anticipated. Genome mining of BGCs in the diversity of the *Planktothrix* revealed a wider potential in the benthic and biphasic life styles that the one seen so far from closely related planktic *Planktothrix*.

Finally, it is also interesting to compare the main characteristics of the planktic *Planktothrix* genomes with those of *Microcystis*, another major toxic bloom-forming cyanobacteria in freshwater ecosystems. On one hand, *Microcystis* genomes are characterized by a very high genomic plasticity featured by the high number of repeated and insertion sequences, and strickingly low synteny value between 12 closely related strains^28^. It was previously hypothesized that *Microcystis* genomic plasticity allowed these species to occupy a wide range of environments^28^. On the other hand, the 11 planktic *Planktothrix* genomes investigated in this study revealed higher synteny values and smaller pan genome. Knowing that planktic *Planktothrix* blooms mainly occur in temperate and cold areas when *Microcystis* blooms are found in all latitudes, these genomic differences might be associated for *Planktothrix* to a smaller ability to develop in various environmental conditions. Interestingly, our genomic data confirm that red and green *Planktothrix* strains belong to the same species and consequently that they can be considered as ecotypes occupying different ecological niches, the red strains being able to bloom in deep and cold lakes when green strains mostly bloom in temperate shallow lakes^22^. The differentiation of ecotypes occupying separate niches has been described in marine picocyanobacteria belonging to the *Prochlorococcus* genus, but the *Prochlorococcus* genomes are characterized by a reduced size while there is no genome size reduction in *Planktothrix*. As proposed by Humbert *et al*.^28^ the larger variability in environmental conditions of freshwater ecosystems in comparison to large ocean areas may require larger adaptive capacities for freshwater cyanobacteria.

Overall, this work opens new perspectives in cyanobacterial studies related to evolution and adaptation and life style duality in single genera. The first report here of a nitrogen-fixing *Planktothrix* strain challenges the commonly known features of this genus and should encourage investigations on distribution and evolution of diazotrophy in the cyanobacterial phylum as well as raise questions on the ecological implications of such finding. The wider potential for natural products of this genus should lead to the survey and monitoring of more potentially hazardous compounds, especially in understudied benthic blooms.

## Methods

### Description of the sequenced strains and of the DNA isolation

Six axenic PCC strains of *Planktothrix* spp. were grown in BG11 media at 18 °C for PCC 7821, and at 22 °C for all others (Table 1). These strains isolated at different geographical locations displayed different life styles in culture conditions such as planktic, biphasic and benthic (Fig. S1). The cultures to obtain biomass for genomic DNA isolation or/and electron microscopy capitation of the gas vesicules are detailed in the supplementary information.

### Estimation of the growth under different nitrogen concentrations

For growth under nitrogen deprivation, actively growing cultures were harvested by gentle centrifugation prior to be transferred in four BG11 differing in nitrate availability (ca 18 mM to 9 mM, 2 mM and none). Three successive transfers were performed. In addition, cultures in BG11o showing depigmentation or discoloration were transferred in BG11_2_ to test their survival with supplemented nitrate. We compared also the effect of light/dark cycle to continuous light on the nitrogen fixation on cultures grown in BG11 and BG11o. Finally, the diazotrophic *Cyanothece* sp. PCC 7822 was included as positive control for nitrogen fixation^39^.

### Sequencing and assembling methods

The whole genome sequences PCC 7805 and PCC 7821 were obtained using next generation sequencing technologies. A 454 library was constructed and around 20-fold coverage of GSflx (www.roche.com) reads were assembled using Newbler (www.roche.com). Details of the assembly are resumed in the supplementary informations. For each genomic DNA of PCC 8927, PCC 9214, PCC 9631 and PCC 11201, a mate-paired library with around 10 kb insert size was performed. The sequencing was realized on Illumina MiSeq device (producing 250 nt length for each fragment extremity) leading to ~150-fold coverage per genome. The read data were assembled using Velvet (https://www.ebi.ac.uk). To reduce the number of undetermined bases, GapCloser (http://soap.genomics.org.cn/soapdenovo.html) was performed on scaffolds with a size >2 kb. The assembly data of all strains were integrated into the Microscope platform for automatic annotation (http://www.genoscope.cns.fr/agc/microscope). *In silico* analyses of the new genomes were performed in MicroScope platform^40^ and resumed in the supplementary information.

### Phylogenetic reconstruction and ANI analysis

The species tree was generated by a concatenation of 586 conserved proteins selected from the phylogenetic markers previously validated for Cyanobacteria^2^,^4^,^41^. Ambiguous and saturated regions were removed with BMGE v1.12^42^ (with the gap rate parameter set to 0.5). A Maximum-Likelihood phylogenetic tree was generated with the alignment using RAxML v7.4.3^43^ with the LG amino acid substitution model. The genomes of *Lyngbya* sp. CCY 9616 and *Arthrospira* sp PCC 8005 were used as outgroup in order to root the phylogenetic tree with the closest relatives as indicated in the extended phylogenetic tree (Fig. S4). The phylogenetic tree was displayed and annotated using the interactive tree of life (iTOL) online tool^44^. *Nif* phylogeny was built on the protein sequences of NifB, NifD, NifH, NifS and NifU and performed with the SeaView package (version 4.6.1)^45^. Briefly, genes were independently aligned using MUSCLE (vers. 3.5)^46^. The alignments were concatenated and filtered to remove all invariable sites. The phylogenetic analysis was performed using PhyML for Maximum Likelihood^47^ with a GTR (generalise time-reversible) substitution model and bootstrap analysis (1000 replicates). The *GvpC* phylogeny was conducted similarly to the *Nif* phylogeny.

Average nucleotide identity (ANI) was computed using the Jspecies package^48^. Whole genome similarity calculation were identical regardless the ANI algorithm used (ANIb based on blast algorithm and ANIm based on MUMmer algorithm).

### Gas vesicle protein C gene analysis

The GvpC sequences of *Planktothrix* were aligned with clustal–omega (http:// www.ebi.ac.uk/Tools/msa/clustalo/). Manual search for repeated structure in each protein sequences was performed by using the consensus 33 amino acid repeats described in GvpC of *Anabaena* sp. (HKdLekdtQeFLSdTAkeRmAQAkeQAeqLhqF) and of *Calothrix* sp. (HKeLqetsQqFLSaTAqaRiAQAekQAqeLlaF)^49^. The repeats of GvpC proteins were analyzed with Weblogo (http://weblogo.berkeley.edu/logo.cgi) and compararison of GvpC was performed by BLAST analysis (http://blast.ncbi.nlm.nih.gov/Blast.cgi).

### Tolytoxin detection

Tolytoxin detection was performed by HPLC-MS and NMR as described for the characterization of this compound in *Scytonema* sp. PCC 10023^21^. NMR spectra were recorded on a Bruker Avance III spectrometer equiped with a cold probe at 500 MHz for ^1^H NMR. Chemical shifts were referenced to the solvent peaks at δ_H_ 2.05 for acetone-*d*_6_. LC-ESI mass spectrometry was performed on a Thermo Scientific Q Exactive mass spectrometer.

## Additional Information

**How to cite this article**: Pancrace, C. *et al*. Insights into the *Planktothrix* genus: Genomic and metabolic comparison of benthic and planktic strains. *Sci. Rep.* **7**, 41181; doi: 10.1038/srep41181 (2017).

**Publisher's note:** Springer Nature remains neutral with regard to jurisdictional claims in published maps and institutional affiliations.

## Supplementary Material

Supplementary Information

## Figures and Tables

**Table 1:** Description of the six *Planktothrix* strains studied.

Strains	Geographical origin and date of isolation	Life style*
*P. agardhii* PCC 7805	Veluwemeer, The Netherland, 1972	Planktic
*P. rubescens* PCC 7821	Gjersjøen lake, Norway, 1971	Planktic
*P. serta* PCC 8927	Berre pond, France, 1988	Benthic
*P. tepida* PCC 9214	Gut of *Culex decens*, Central African Republic, 1989	Biphasic
*P. paucivesiculata* PCC 9631	Marne river, France, 1996	Benthic
*Planktothrix* sp. PCC 11201	Waitaki river, New Zealand, 2012	Benthic

^*^See Fig. S1.

**Table 2:** General features and accession numbers of the six *Planktothrix* genomes.

Strains	Statut*		Contigs AC	Genome size (bp)	GC%	No of scaffolds	No of contigs	CDS	Average CDS length (bp)	misc_RNA	rRNA	tRNA
*P. agardhii* PCC 7805	Complete	Chr.	LO018304	4,700,732	39.62	1	1	4,296	937	14	3	40
		MP	LO018305	151,88	39.87	1	1	150	902	/	/	/
		P	LO018306	4,522	38.35	1	1	5	522	/	/	/
*P. rubescens* PCC 7821		Chr.	CZCZ01000001-08	5,391,870	39.47	5	8	4,884	951	18	1	40
	WGS	MP	CZCZ01000009-12	196,481	39.01	4	4	178	941	/	/	/
		P	CZCZ01000013	12,935	41.04	1	1	18	511	/	/	/
*P. serta* PCC 8927	WGS	/	CZCU01000001-178	6,250,174	39.58	157	178	5,729	940	12	3	42
*Planktothrix* sp. PCC 11201	WGS	/	CZCT01000001-151	5,964,975	40.14	103	151	5,202	1,002	17	6	48
*P. tepida* PCC 9214	WGS	/	CZDF01000001-209	6,725,787	39.47	191	209	6,043	957	33	3	48
*P. paucivesiculata* PCC 9631	WGS	/	CZCS01000001-244	6,072,585	40.10	184	244	5,365	994	18	6	48

**Table 3:** Distribution and diversity of the gene clusters involved in the biosynthesis of natural products of the studied *Planktothrix*.

	RiPPs					NRPS					NRPS/PKS hybrid					PKS				
	*mdn*	pat-like	*osc*	*pag*	RiPP1	*aer*	*apt*	*oci*	PNL2	PNL3	*mcy*	*mic*	*has*-like	PNL1	PHL1	*lum*	*tto*	PPL1	PPL2	PPL3
Size (kb)	7,1–7,3	11,1	8,2–11,8	9,3–11,4	3,4	22,8–25,7	23,7	27,5–32,3	11	11,3	49,3–51,1	34,5	43,2	25,1	28	89,5	92,8	11,7	8	6,2
PCC 7821	Ref.	—	Ref.	Ref.	—	Ref.	Ref.	Ref.	100^£^	—	Ref.	100^£^	—	—	—	—	—	—	—	—
PCC 7805	93	—	97	96	—	82	*part*E	91	—	—	—	—	—	96^£^	—	—	—	—	—	—
PCC 11201	92	—	83	92 + *part*ABC	—	54	—	55	—	—	73	—	—	—	—	—	76^§^	—	—	—
PCC 9214	—	—	—	—	—	—	*part*BCD	—	—	Ref.	—	—	—	—	Ref.	—	—	—	Ref.	Ref.
PCC 8927	—	—	—	—	Ref.	—	—	—	—	—	—	—	66^$^	—	—	—	—	Ref.	—	—
PCC 9631	—	91*	—	*part*GEF	—	—	—	—	—	—	—	—	—	—	—	Ref.	—	—	—	—

Ribosomally synthesized and post-translationally modified peptides (RiPPs), non-ribosomal peptide synthetase (NRPS), polyketide synthase (PKS) and NRPS/PKS hybrids were compared by amino acid identity (% AAI) of NRPS/PKS/cyanobactin protease to a reference *Planktothrix* strain (Ref.), *Microcystis* sp. PCC 9432 (*), *Anabaena* sp. SYKE748A ($), *Planktothrix prolifica* NIVA-CYA 98 (£) and *Scytonema* sp. PCC 10023 (§). Incomplete gene clusters are indicated with the conserved genes (*part*). The biosynthetic gene clusters of aeruginosin (*aer*), anabaenopeptin (*apt*), cyanopeptolin (*oci*), luminaolide (*lum*), microcyclamide (*mca*), microcystin (*mcy*), microginin (*mic*), microviridin (*mdn*), oscillatorin (*osc*), prenylagaramide (*pag*), tolytoxin (*tto*) were detected as well as one like hassallidin (*has*-like), and another one like cyanobactin (*pat*-like), and other *Planktothrix* gene clusters not corresponding to know molecules named *Planktothrix* NRPS-like (PNL), *Planktothrix* PKS-like (PPL) and *Planktothrix* hybrid-like (PHL).
