# Supplementary material for: Insights into the Planktothrix genus: Genomic and metabolic comparison of benthic and planktic strains
Source: Sci Rep. 2017 Jan 24;7:41181. doi: 10.1038/srep41181 (PMC5259702; doi:10.1038/srep41181)
Supplement: Supplementary Information [file srep41181-s1.pdf]

Insights into the *Planktothrix* genus: Genomic and metabolic comparison of benthic and planktic strains

Claire Pancrace[1,2,3], Marie-Anne Barny[2], Reiko Ueoka[4], Alexandra Calteau[5], Thibault Scalvenzi[1], Jacques Pedron[2], Valérie Barbe[6], Joern Piel[4], Jean-François Humbert[2]*, Muriel Gugger[1]*

[1]Institut Pasteur, Collection des Cyanobactéries, 28 rue du Dr Roux, 75724 Paris Cedex 05, France. [2]UMR UPMC 113, CNRS 7618, IRD 242, INRA 1392, PARIS 7 113, UPEC, IEES Paris, 4 Place Jussieu, 75005, Paris, France. [3]Université Pierre et Marie Curie (UPMC), 4 Place Jussieu, 75005, Paris, France. [4]Institute of Microbiology, Eigenössische Technische Hochschule (ETH) Zurich, Vladimir-Prelog-Weg 4, 8093 Zurich, Switzerland. [5]Commissariat à l'Energie Atomique et aux Energies Alternatives (CEA), Genoscope & CNRS, UMR 8030, Laboratoire d'Analyse Bioinformatique en Génomique et Métabolisme, 2, rue Gaston Crémieux, CP 5706, 91057 EVRY cedex, France. [6]Commissariat à l'Energie Atomique et aux Energies Alternatives (CEA), Genoscope, Laboratoire de Biologie Moleculaire pour l'Etude des Génomes, 2, rue Gaston Crémieux, CP 5706, 91057 EVRY cedex, France. *Correspondence and requests for materials should be addressed to JFH (jean-francois.humbert@upmc.fr) or MG (mgugger@pasteur.fr).

Supplementary material

Methods

Cultivation for biomass and DNA isolation. For DNA isolation, five strains were grown at 25 °C in BG11 medium buffered with 5 mM of $NaHCO_3$ in 2L Erlenmeyer flask with bubbling 1% CO_2, agitated by magnetic bar with continuous light, whereas the strain PCC 7821 was grown at 18°C without agitation and exposed to a 13h-11h light-dark cycle at 20 µmol photon.m^{-2}.s^{-1}. They were harvested by centrifugation (10,000 g, 10 min, 18°C), washed twice with sterile distilled water, and kept frozen until DNA extraction. DNA extraction of the frozen pellets was carried out using Genomic DNA isolation-NucleoBond H AX (Macherey-Nagel, Hoerdt, France) according to the manufacturer's instructions.

Media for nitrogen deprivation and growth conditions. BG11 contains 17.67 mM of $NaNO_3$ and corresponds to the medium used for maintaining and transferring

Planktothrix strains each month at the PCC. The other BG11-based media used were: BG11o, medium with no combined nitrogen source; $BG11_9$ with 9 mM of $NaNO_3$ and $BG11_2$ with 1.8 mM of $NaNO_3$. All the cultures were grown at 22°C under a rhythm of 13h-11h light-dark cycle at 20 µmol photon.m^{-1}.s^{-2}. Each culture was grown for 1 month before being transferred (dil. 1/20 for planktic form or a fragment of the biofilm for the benthic form) into a subculture in the same conditions. The growth and the pigmentation of the strains were estimated visually due to the fact that depending on the life style (benthic *versus* planktic), the filaments were more or less aggregated in pellet or in biofilms (Fig. S1), making the estimation of the optical density impossible.

Genome assembly. Assembly validation was made via the Consed interface (www.phrap.org), and 287 and 494 PCR reads for PCC 7805 and PCC 7821, respectively, were performed for gap closure. For the quality assessment, around 100-fold coverage of Illumina reads (GAIIX instrument, 51 bp) were mapped onto the whole genome sequences, using SOAP (http://soap.genomics.org.cn), as described by Aury *et al.*[1]. Additionally as tRNA histidine was missing from the original assembly of our two planktic genomes (the same way, tRNA isoleucine is lacking in the genomes of eight available planktic *Planktothrix*), we resequenced their genomes (Illumina NextSeq500 technology) to find tRNA histidine located between highly repetitive sequences.

Core and pan-genome. The Pan/Core genome functionality of the MicroScope platform was used to compute the core and the pangenome of *Planktothrix* strains[2]. Putative orthologs were defined as gene pairs satisfying an alignment threshold of at least 80% amino acid sequence identity over at least 80% of the length of the smallest protein.

Phylogenetic tree reconstruction. The extended species tree was generated by a concatenation of twenty-nine conserved proteins selected from the phylogenetic markers previously validated for Cyanobacteria[3]. A Maximum-Likelihood phylogenetic tree was generated with the alignment using PhyML 3.1.0.2 using the LG amino acid substitution model with gamma-distributed rate variation (six categories), estimation of a proportion of invariable sites and exploring tree topologies using Nearest Neighbor Interchanges.

Synteny and estimation of the proportion of repeated sequences. Synteny computation and repeated sequence detection are provided by the MicroScope platform. The proportion of repeats was estimated using the Repseek algorithm, a fast two-step method (seed detection followed by their extensions), which allows finding large

degenerate repeats within or between large DNA sequences[4]. The synteny values representing the percentage of CDSs belonging to a synteny group were estimated by taking into account CDSs sharing at least 35% sequence identity on 80% of the length of the smallest protein, with a gap parameter (number of consecutive genes not involved in synteny) set to five.

Comparative analysis of the distribution of the genes among the COG categories. For each genome, all genes were assigned to a COG category by using the tool COGnitor available in MicroScope platform, knowing that COGnitor compares the gene sequences to the *COG* database by using BLASTP[5]. A non-metric multidimensional scaling (NMDS) was performed on the relative abundances of each COG category in all *Planktothrix* genomes available.

Detection of natural product gene clusters in *Planktothrix* strains. Natural product gene clusters were identified using the antiSMASH 2.0.2 software[6] and the modified version of the complete genome scanning pipeline 2metdb[7]. Each gene within a cluster was compared to its syntenic homolog at the amino-acid level in the reference genome such as *P. rubescens* PCC 7821 to obtain the deduced amino-acid sequence identity (AAI). Features of genomic plasticity were identified using RGPfinder with SIGI-HMM[8] and AlienHunter (IVOM)[9] incremented on the MicroScope platform.

Microscopy protocol for TEM of the gas vesicles. Cell material (15 mL of one month old culture) was centrifuged for 30 min at 3800g at room temperature. The cell pellets were fixed overnight in Sörensen buffer (Na_2PO_4/NaH_2PO_4 0,1M pH 7,2) supplemented with sucrose 0,18M, glutaraldehyde 2% (vol/vol) and paraformaldehyde 2% (vol./vol.). Fixed samples were rinsed three times with Sörensen buffer, post-fixed for 1 h at room temperature with 1% (wt/vol) osmium tetraoxide in Sörensen buffer, and rinse three times in Sörensen buffer. The samples were then dehydrated at room temperature through a graded series of 30 min ethanol baths (ethanol/ Sörensen buffer 30%, 50%, 70%, 90%, 100%, vol./vol.). Dehydrated samples were further embedded in a graded series of ultrabed low viscosity resin baths of 30 min each (resin/ethanol 25%, 33%, 50%, 100%, vol./vol.). The last bath in 100% low viscosity resin was repeated 3 times and samples were then polymerized 48h at 37°C.

Figure S1. Three weeks-old cultures of the six *Planktothrix,* steady (A) or agitated (B) and their life style.

A PCC 7805 PCC 7821 PCC 9214 PCC 11201 PCC 9631 PCC 8927

B

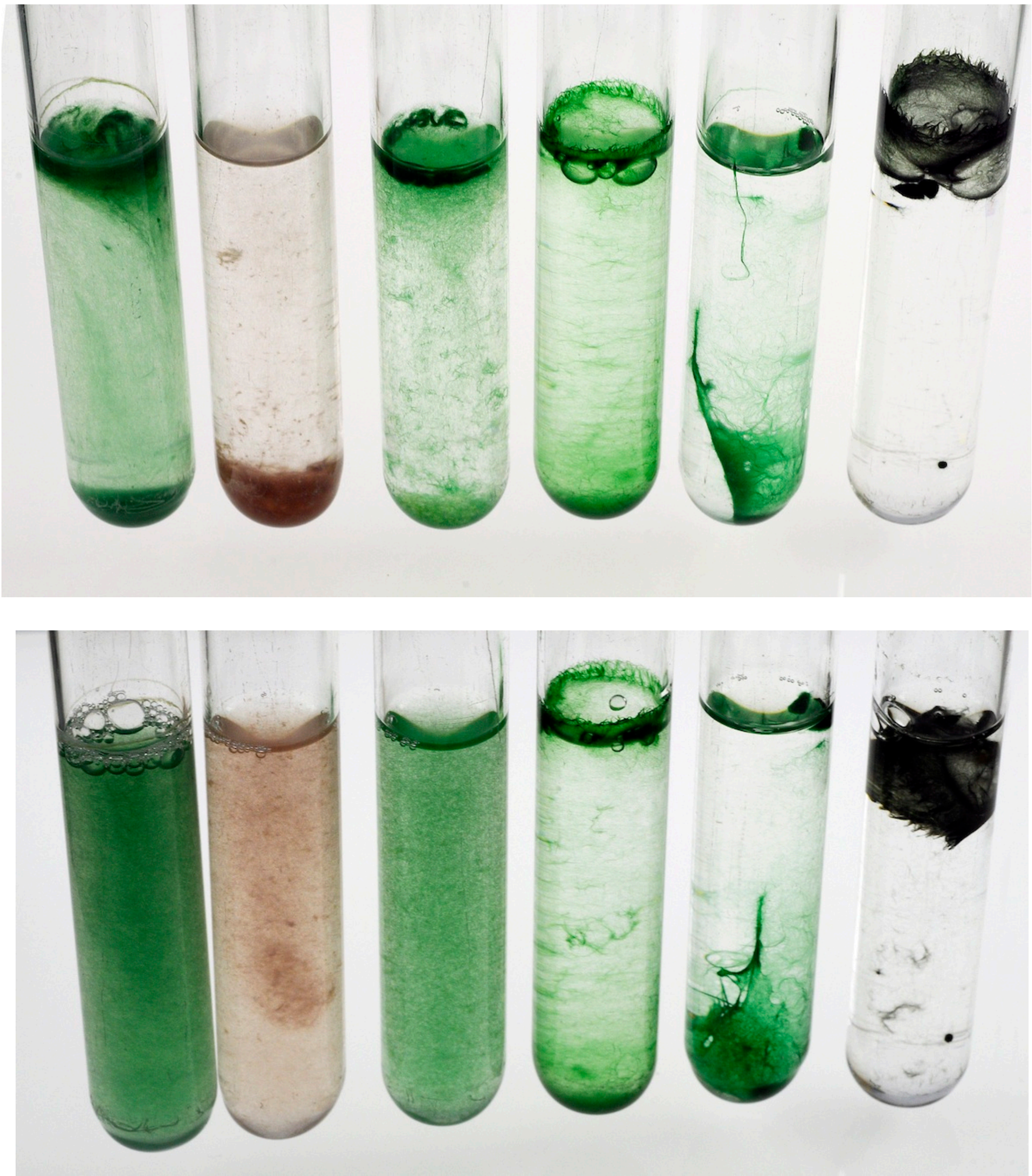

	PCC 7805	PCC 7821	PCC 9214	PCC 11201	PCC 9631	PCC 8927
Origin	Temperate lake	Nordic lake	African lake - insect gut	River	River	Waste water tank
Biofilm formation and attachment				Biofilm attached on wall surface	Biofilm tightly attached to the bottom of the flask	Biofilm loosely attached to wall at air/water interface
Life style of the isolated material	Planktic (water column)	Planktic (water column)	Unknown	Benthic mat	Benthic	Unknown
Strain life style in culture	Planktic	Planktic	Biphasic, depends on culture conditions	Benthic	Benthic	Benthic

Figure S2. Percentage of CDSs belonging to a synteny group in 15 *Planktothrix* genomes and *Lyngbya* sp. CCY9616.

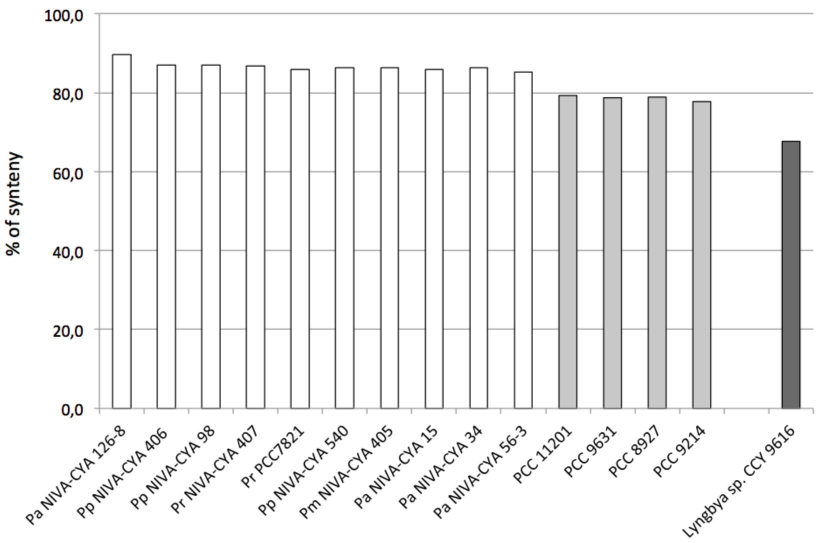

Figure S3. NMDS analysis of COG of 15 *Planktothrix* genomes.

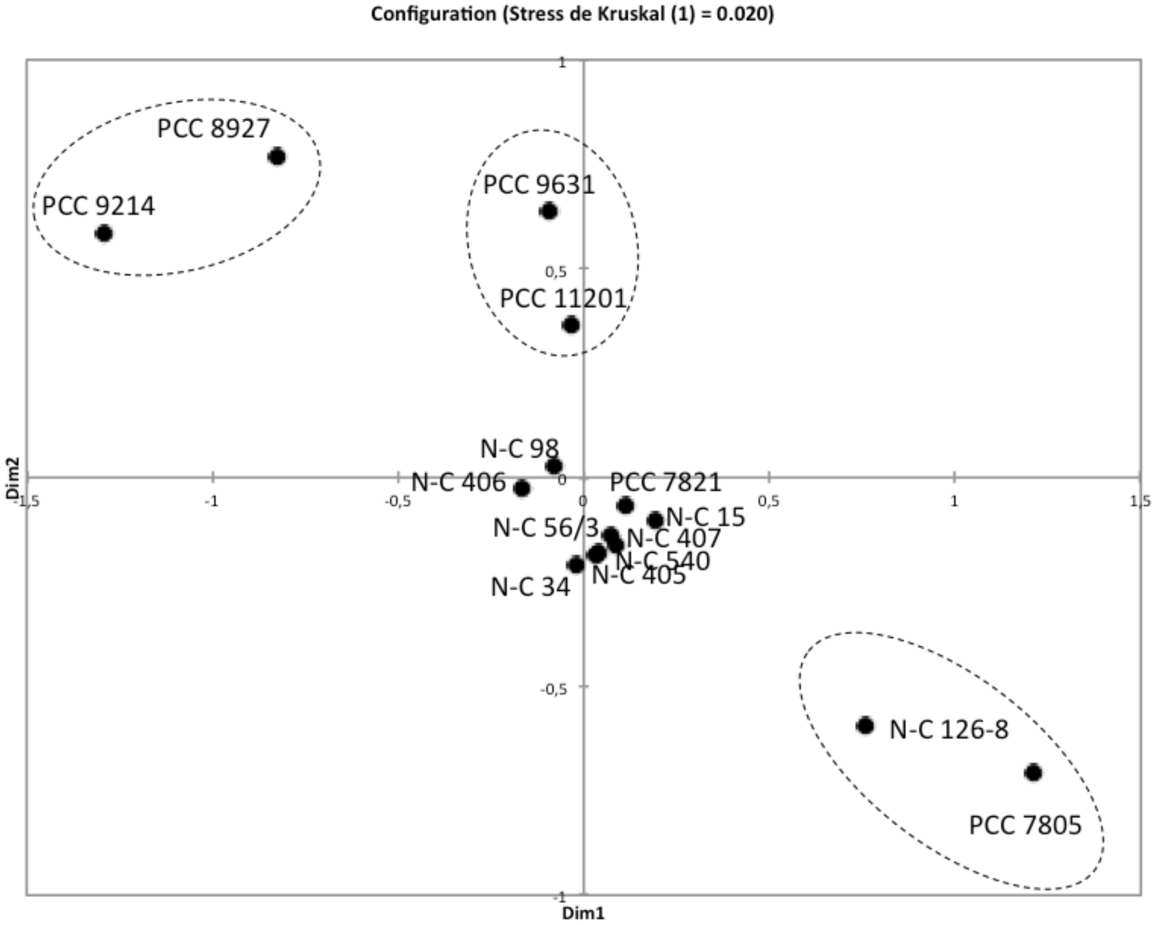

Figure S4. Extended phylogeny of the *Planktothrix* clade with their closest relatives (*Arthrospira* sp. PCC 8005 (GCA_000973065.1), *Arthrospira maxima* CS-328 (ABYK00000000), *Arthrospira platensis* NIES-39 (GCA_000210375.1), *Lyngbya* sp. CCY 9616 (AAVU00000000), *Oscillatoria* sp. PCC 10802 (ANKO00000000), *Oscillatoria acuminata* PCC 6304 (CP003607-09), *Oscillatoria nigro-viridis* PCC 7112 (CP003614-19), *Microcoleus vaginatus* FGP-2 (AFJC00000000), *Oscillatoria* sp. PCC 6506 (CACA00000000), *Oscillatoria formosa* PCC 6407 (ALVI00000000), *Trichodesmium erythraeum* IMS101 (CP000393.1)). The species tree was generated by a concatenation of twenty-nine conserved proteins (DnaG, Frr, NusA, Pgk, PyrG, RplA, RplB, RplC, RplD, RplE, RplF, RplK, RplL, RplM, RplN, RplP, RplS, RplT, RpmA, RpoB, RpsB, RpsC, RpsE, RpsI, RpsJ, RpsK, RpsM, RpsS and SmpB) using a Maximum Likelihood method[3]. Bootstrap values superior or equal to 70% are indicated.

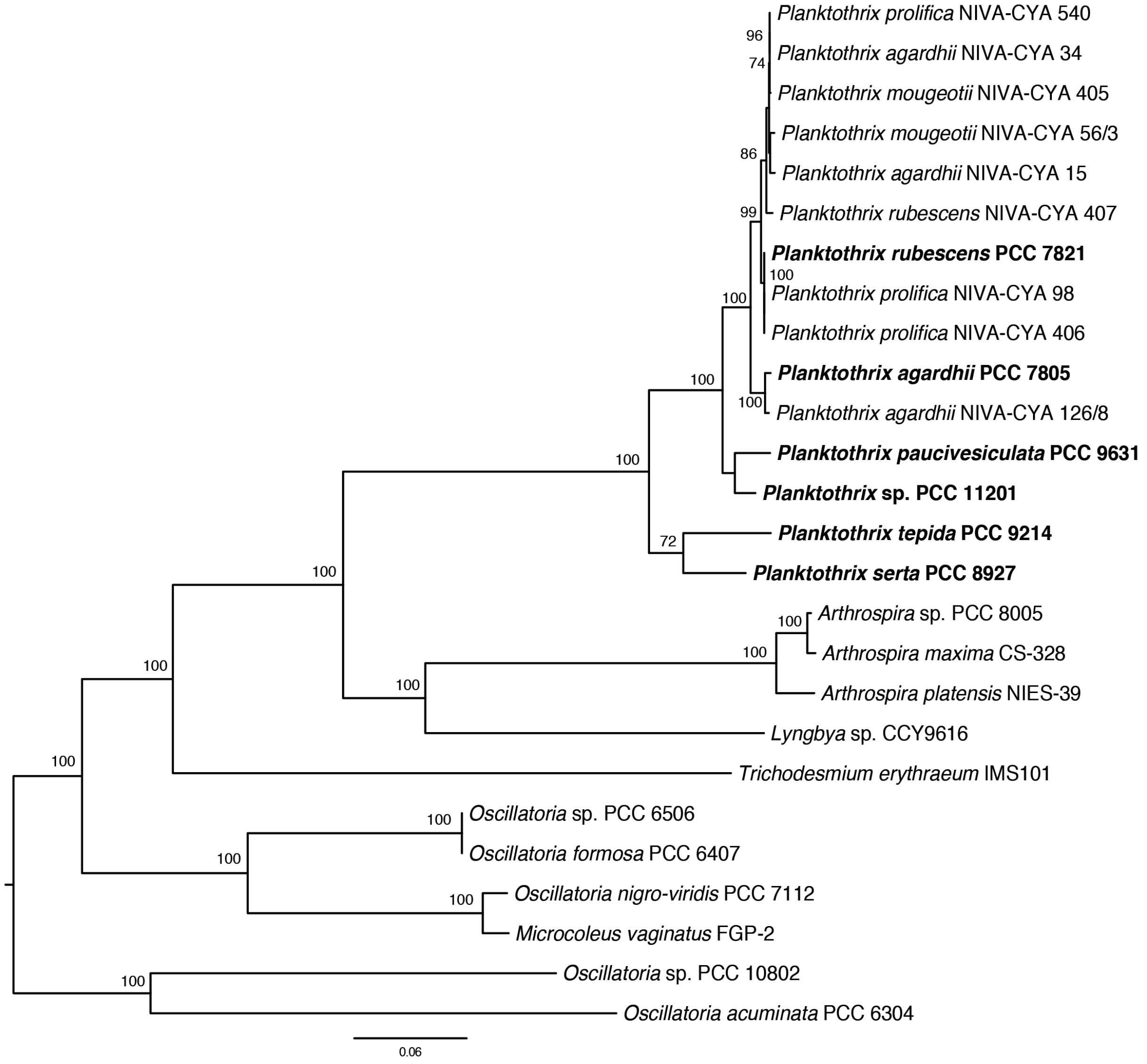

Figure S5. Diversity of the protein GvpC using a Maximum Likelihood method. The benthic strains are indicated in bold.

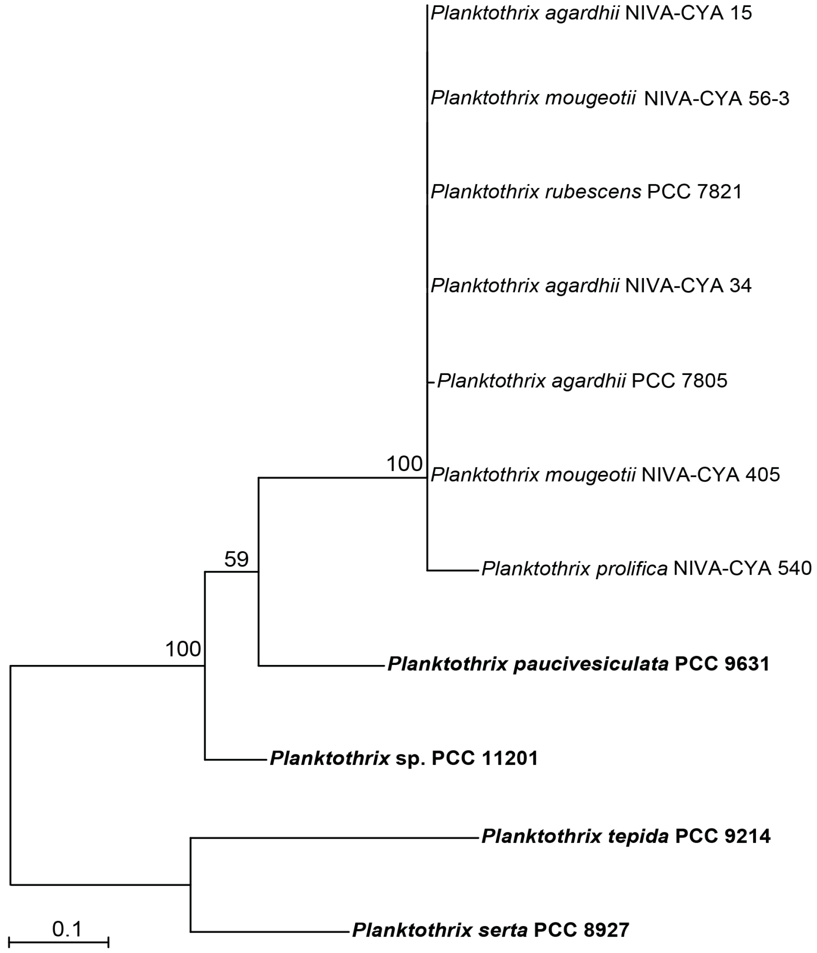

Figure S6. Transmission electronic microscopy photographies of *Plankthotrix* sp. PCC 11201 (upper pictures) and *P. agardhii* PCC 7805 (lower pictures) after a month of culture. The scale bars are expressed in nm.

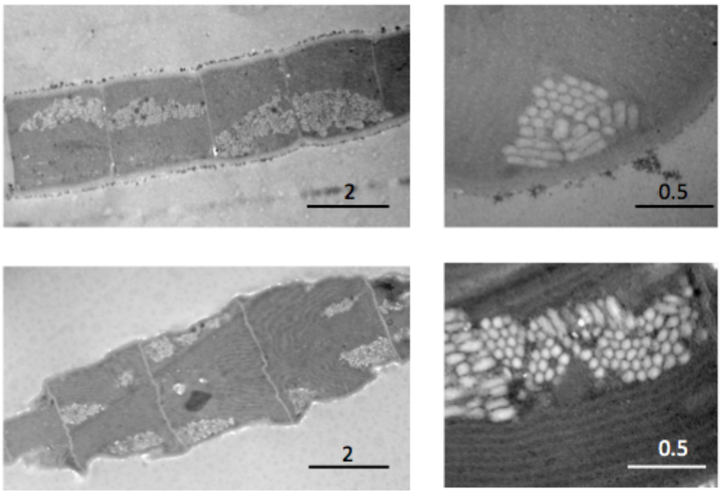

Figure S7. Phylogenetic tree constructed by Maximum Likelihood on the concatenated sequences of *nifBDHSU* genes. The *Planktothrix* are indicated in bold.

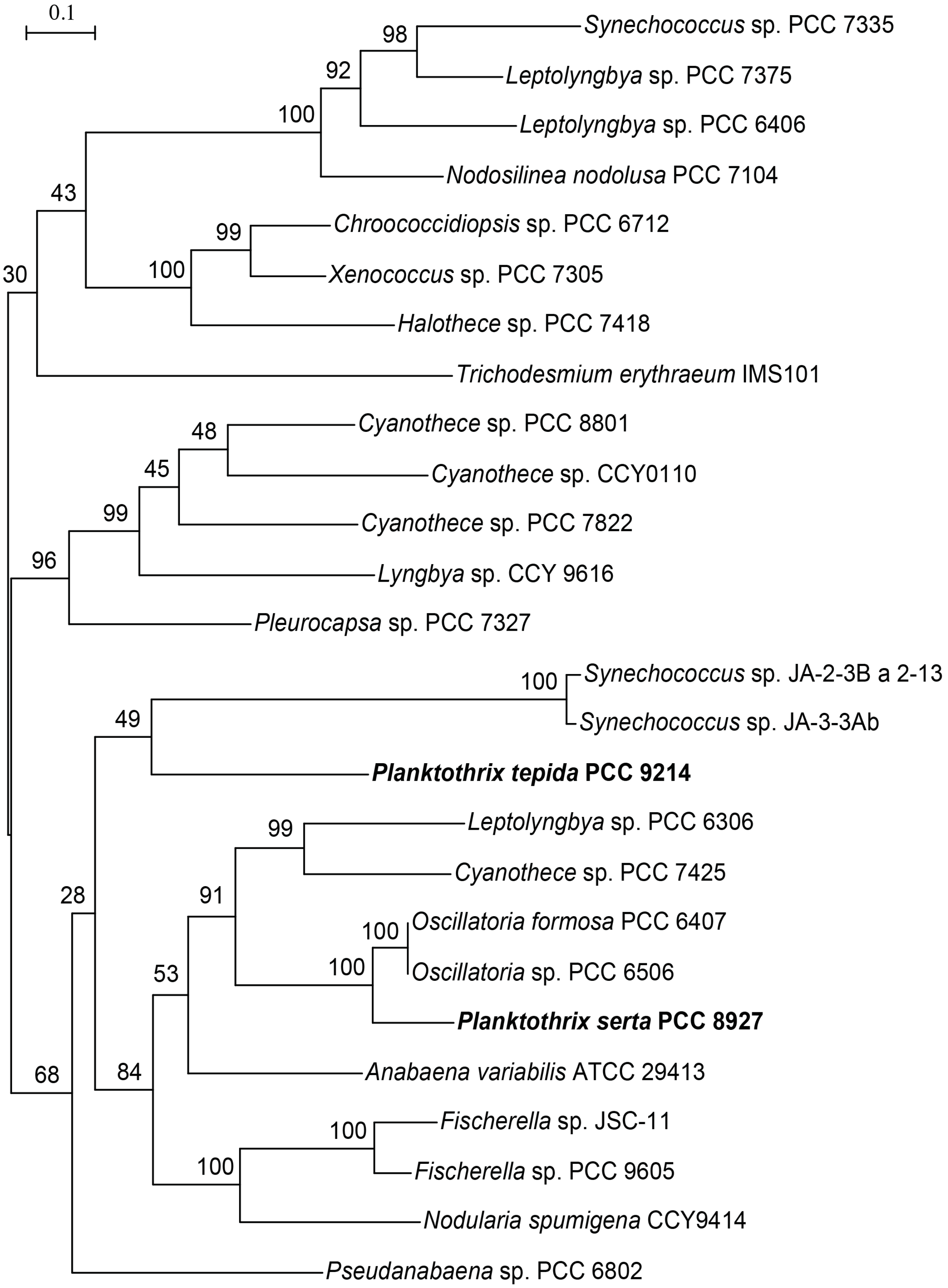

Figure S8. *Planktothrix* nitrate tolerance and nitrogenase activity in *P. serta* PCC 8927. Cultures of three successive transfers (GN1, GN and GN3) of *Planktothrix* are compared to the diazotroph *Cyanothece* sp. PCC 7822. The cultures observed after a month showed different aspect in function of the nitrate present in the medium; either they grew well with nice expected pigmentation (black); or grew with attenuated pigmentation (grey); or grew with a clear depigmentation, or showed very little growth with still some pigmentation (grey with P) or no growth at all (white). The pictures show the second or third transferred cultures from left to right in BG11, in $BG11_9$, $BG11_2$, and BG11o for PCC 7805 (A), PCC 7821 (B), PCC 8927 (C), PCC 9214 (D), PCC 9631 (E), PCC 11201 (F), PCC 7822 as control of nitrogenase activity (G), and the cultures of the first transfer in BG11o were transferred in $BG11_2$ to check survival, only PCC 7821 died irreversibly (H).

Strains	nif gene cluster	lifestyle	GN1	GN2	GN3	GN1	GN2	GN3	GN1	GN2	GN3	GN1	GN2	GN3
			18 mM nitrate			9 mM nitrate			2 mM nitrate			0 mM nitrate		
PCC 7805	-	planktic									P	P		
PCC 7821	-	planktic										P	P	
PCC 8927	+	benthic												
PCC 9214	+	biphasic			P							P	P	
PCC 9631	-	benthic										P	P	
PCC 11201	-	benthic										P	P	
Control (+)	+													

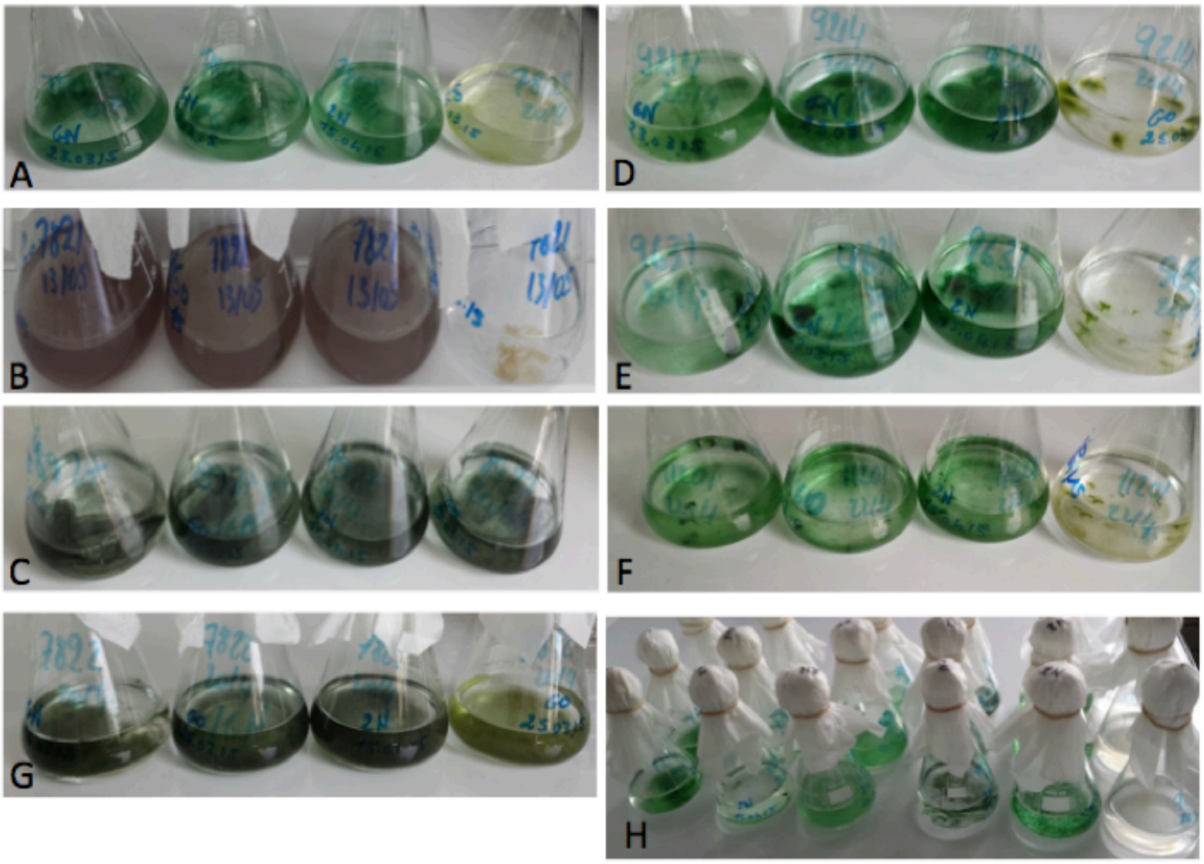

Figure S9. NMDS (Jaccard Indexes) on the distribution of natural product biosynthetic gene clusters in *Planktothrix*

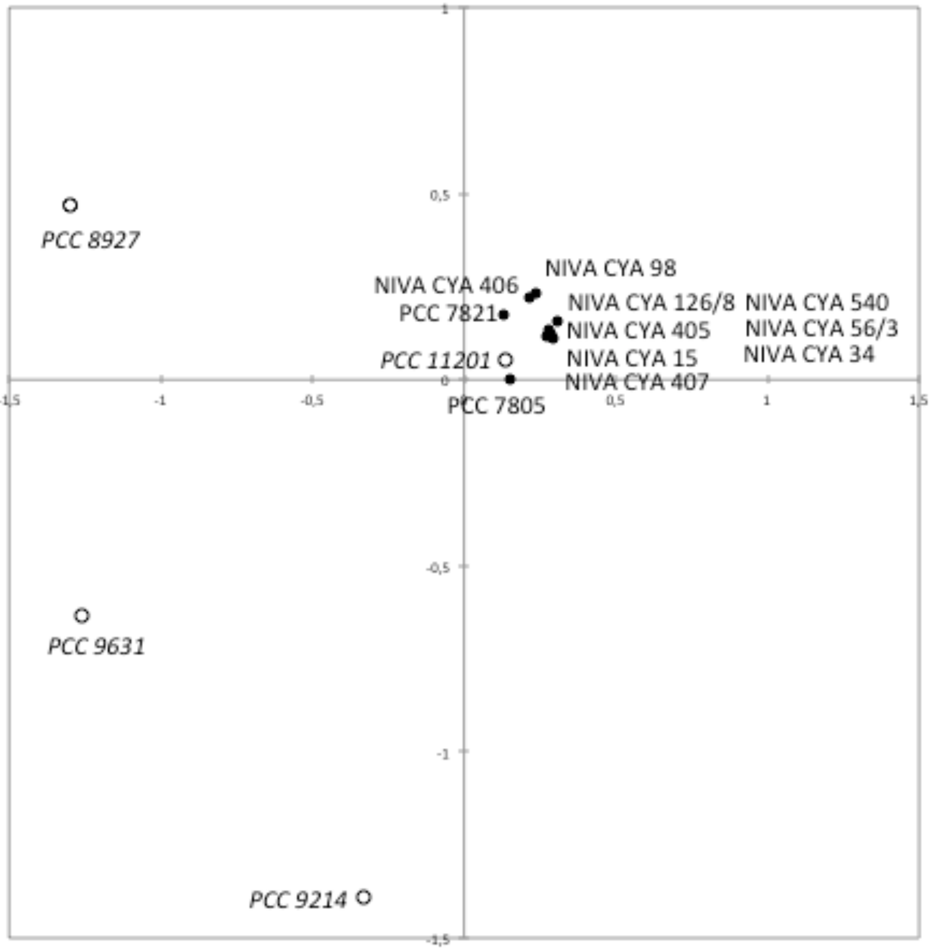

Figure S10. *Planktothrix* sp. PCC 11201 produces tolytoxin.

A. ^{1}H NMR data of tolytoxin from PCC 10023 (upper) and PCC 11201-1 (lower) in acetone-d_6 at 298 K; B. HR-LCMS data of the extracts of PCC 11201. Extracted ion chromatogram (*m/z* 872.50-872.52) of tolytoxin obtained from PCC 10023 (upper) and the extract of PCC11201 (middle); and mass spectrum of the peak at 17.69 min (*m/z* 872.51 $[M+Na]^+$, *m/z* 832.52 $[M+H-H_2O]^+$, *m/z* 814.51 $[M+H-2H_2O]^+$).

A

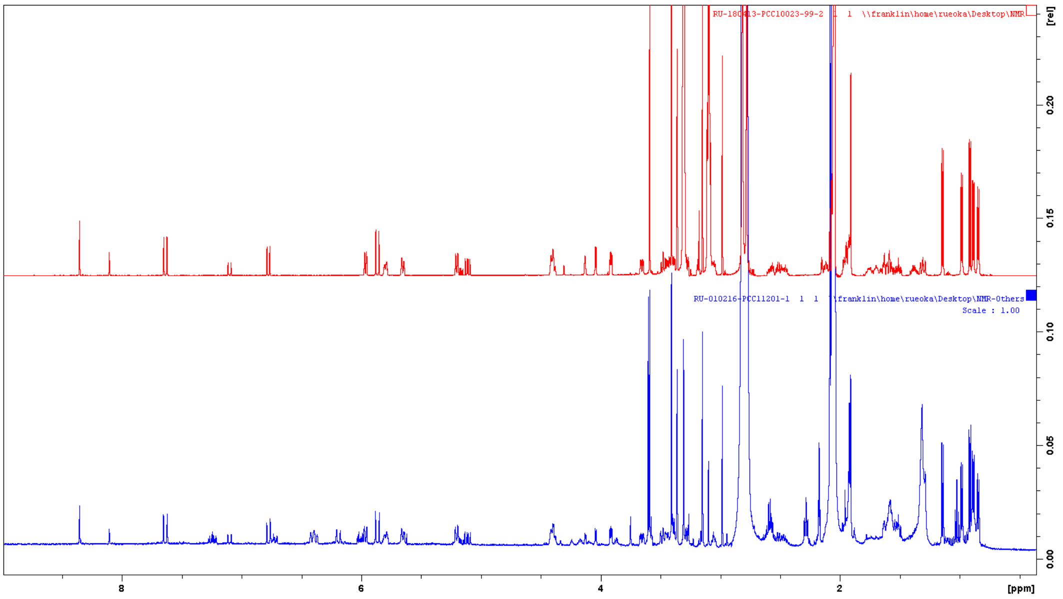

B

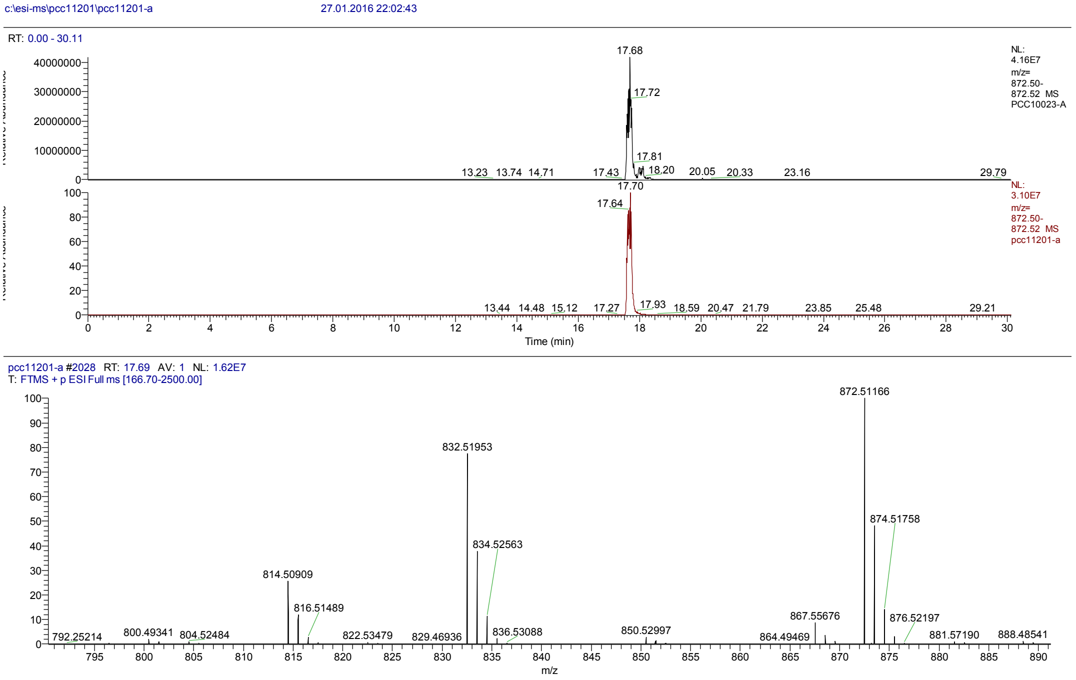

Figure S11. Cyanobactin gene cluster *pat* in the strain *Planktothrix paucivesiculata* PCC 9631. (A) Comparison with conserved gene cluster in other strains producing different cyanobactins. Genes colors indicate protein function in cyanobactin biosynthesis: N-terminal protease in black, heterocyclase in light blue, precursor in dark blue, methyltransferase in grey, putative prenyltransferase in orange, C-terminal protease and oxidase in red. Genes in green and white code proteins of unknown function respectively involved or putatively involved in cyanobactin biosynthesis. (B) Alignments of cyanobactin precursors peptides. Core sequences are framed.

A

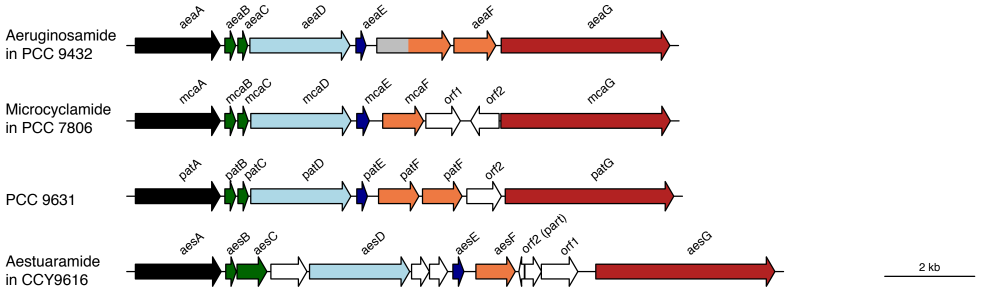

B

Microcyclamide	MDKKNLLPNQGAPVIRGISGKLPSHLAELSEEALGGNGAEAS	ATVSIC	AFDGAEAS	FTGCMC	AFDGAEAS	ITGCIC	AFDGAEAS	ITGCIC	AFDGDEA
Aeruginosamide	MDKKNILPHQGKPVLRTTNGKLPSHLAELSEEALGGAGMDAS			FFPC	SYDGADAS	FFPVC	SYDGADAS	FFPC	SYDDGDA
Unknown	MDKKNILPHQGKPVLRSTNGKLPSHLAELSEEALGGNGVDAS			MCPCVC	SYDGVDAS	MCPCVC	SYDGVDAS	MCPCVC	SYDGDE
Aestuaramide	MDKKNILPHQGKPVLRTTNGKLPSHLAELSEEALGGNGVDAS			ACMPCYP	SYDGVDAS	VCMPCYP	SYDGVDAS	VCMPCYP	SYDDAE

Figure S12. Extended microginin biosynthetic gene clusters.

Green genes were previously described in *mic* gene cluster while the red genes are additional genes to be included in the gene cluster. Yellow genes correspond to transposases and inactivated derivatives. Flanking genes non relevant to the gene cluster are in white. Double oblic bars indicate contig limit. Grey areas connect genes with conserved sequence. ORF1 encodes a CAL domain, ORF2 encodes a peptidyl carrier protein domain, ORF3 encodes an *O*-methyltransferase, while ORF4 is a conserved gene of unknown function. The extended gene cluster in PCC 7821 share 100% AAI with NIVA-CYA 98 and 73% AAI with PCC 9432.

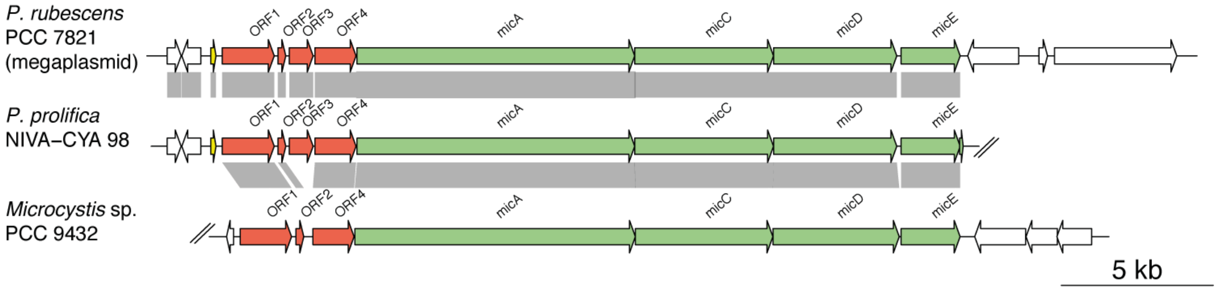

Table S1. COG of the *Planktothrix* genomes

		Strain	PCC 8927	PCC 9214	PCC 9631	PCC 11201	PCC 7821	PCC 7805	N-C 15	N-C 34	N-C 56/3	N-C 126-8	N-C 405	N-C 98	N-C 406	N-C 540	N-C 407
	COG	Total CDSs classified in at least one COG group	66.05%	64.06%	64.54%	65.82%	66.07%	67.10%	67.21%	66.30%	66.94%	65.47%	64.97%	65.14%	64.45%	66.46%	66.57%
INFORMATION STORAGE AND PROCESSING	A	RNA processing and modification	-	-	-	-	0.0203 %	-	-	-	-	-	-	0.0193 %	0.0189 %	-	-
	B	Chromatin structure and dynamics	0.0349 %	0.0496 %	0.0186 %	0.0192 %	0.0203 %	0.0231 %	0.0206 %	0.0200 %	0.0202 %	0.0216 %	0.0196 %	0.0193 %	0.0189 %	0.0201 %	0.0203 %
METABOLISM	C	Energy production and conversion	4.0453 %	4.0668 %	3.8196 %	3.9731 %	4.1346 %	4.3177 %	4.1967 %	4.0352 %	3.9984 %	4.1164 %	3.9554 %	4.0797 %	4.0091 %	4.1750 %	4.1235 %
	E	Amino acid transport and metabolism	4.8997 %	4.9760 %	4.7326 %	4.9712 %	5.3304 %	5.6800 %	5.4721 %	5.4535 %	5.4725 %	5.7759 %	5.3652 %	5.1624 %	5.1437 %	5.4998 %	5.3829 %
	F	Nucleotide transport and metabolism	1.2729 %	1.3060 %	1.3788 %	1.4971 %	1.4795 %	1.6393 %	1.5017 %	1.5581 %	1.5145 %	1.5733 %	1.4882 %	1.4695 %	1.3994 %	1.5054 %	1.5438 %
	G	Carbohydrate transport and metabolism	3.7663 %	3.5047 %	3.3352 %	3.4549 %	3.6684 %	3.9714 %	3.6618 %	3.7155 %	3.8166 %	3.8362 %	3.6225 %	3.6156 %	3.5741 %	3.6933 %	3.8391 %
	H	Coenzyme transport and metabolism	2.5458 %	2.5955 %	2.6085 %	2.8983 %	2.9388 %	3.2787 %	3.1269 %	2.9764 %	2.9887 %	3.2759 %	2.9763 %	2.8422 %	2.8177 %	3.0309 %	2.9657 %
	I	Lipid transport and metabolism	1.4996 %	1.7193 %	1.3974 %	1.3628 %	1.3377 %	1.4085 %	1.5635 %	1.5182 %	1.4742 %	1.4655 %	1.4882 %	1.3921 %	1.3616 %	1.5456 %	1.4219 %
	P	Inorganic ion transport and metabolism	3.6269 %	3.6535 %	3.6519 %	3.7428 %	3.9927 %	4.1792 %	4.1144 %	4.2549 %	4.0590 %	3.8147 %	4.1316 %	3.7896 %	3.7632 %	4.2553 %	4.0016 %
	Q	Secondary metabolites biosynthesis, transport	2.5458 %	3.0749 %	2.5899 %	2.7639 %	2.7361 %	2.6784 %	2.7566 %	2.8166 %	2.6656 %	2.8879 %	2.7805 %	2.9196 %	2.9501 %	2.7700 %	2.8032 %
INFORMATION STORAGE AND PROCESSING	J	Translation, ribosomal structure and biogenesis	3.1212 %	2.9757 %	3.1116 %	3.2054 %	3.5671 %	3.9945 %	3.6412 %	3.5757 %	3.5541 %	3.7069 %	3.3875 %	3.5383 %	3.4985 %	3.5126 %	3.5547 %
	K	Transcription	4.2546 %	3.9841 %	4.3972 %	4.3954 %	3.9116 %	3.6943 %	3.6618 %	3.7755 %	3.7561 %	3.7716 %	3.6616 %	3.9637 %	3.9145 %	3.7334 %	3.9001 %
	L	Replication, recombination and repair	6.7480 %	6.4143 %	7.0430 %	6.7946 %	6.7491 %	5.5876 %	6.8093 %	5.7531 %	6.3813 %	6.2716 %	6.0309 %	6.9992 %	6.7322 %	6.1823 %	6.2970 %
POORLY CHARACTERIZED	R	General function prediction only	13.1822 %	12.6467 %	12.8004 %	12.2073 %	12.9510 %	13.1840 %	13.0220 %	13.0443 %	12.8635 %	12.9526 %	12.6101 %	12.7417 %	12.5756 %	12.9065 %	13.1830 %
	S	Function unknown	8.0384 %	6.5135 %	6.7822 %	7.7159 %	7.9246 %	7.8273 %	7.7967 %	7.9704 %	8.0170 %	7.1552 %	7.7541 %	7.6373 %	7.4887 %	7.9486 %	8.3486 %
CELLULAR PROCESSES AND SIGNALING	D	Cell cycle control, cell division, chromosome	1.7088 %	1.5870 %	1.7328 %	1.7083 %	1.6619 %	1.7317 %	1.5635 %	1.6380 %	1.4338 %	1.6595 %	1.5273 %	1.6628 %	1.6074 %	1.6259 %	1.6047 %
	M	Cell wall/membrane/envelope	5.1962 %	5.0091 %	4.9935 %	5.2399 %	5.2290 %	5.5645 %	5.4927 %	5.2537 %	5.4523 %	5.3664 %	5.1302 %	5.1044 %	5.0303 %	5.1786 %	5.1798 %
	N	Cell motility	1.3775 %	1.3887 %	1.6583 %	1.5547 %	1.5809 %	1.2930 %	1.6046 %	1.5382 %	1.6761 %	1.1853 %	1.5273 %	1.4888 %	1.4183 %	1.5656 %	1.5844 %
	O	Posttranslational modification, protein	3.5222 %	3.3890 %	3.4470 %	3.6660 %	3.7495 %	4.0406 %	3.7029 %	3.7555 %	3.7359 %	3.8793 %	3.6029 %	3.6543 %	3.6498 %	3.6933 %	3.6969 %
	T	Signal transduction mechanisms	7.1142 %	7.9021 %	7.7697 %	7.5624 %	6.4653 %	5.5876 %	6.6242 %	6.6720 %	6.7447 %	5.3233 %	6.6771 %	6.1292 %	6.0703 %	6.7644 %	6.3782 %
	U	Intracellular trafficking, secretion, and vesicular	1.1334 %	1.2564 %	1.2111 %	1.2476 %	1.1958 %	1.2468 %	1.2755 %	1.2785 %	1.2520 %	1.2716 %	1.2336 %	1.1214 %	1.1346 %	1.3248 %	1.2594 %
	V	Defense mechanisms	1.3426 %	1.5044 %	1.4161 %	1.5163 %	1.6619 %	1.7086 %	1.4606 %	1.6181 %	1.6155 %	1.4440 %	1.5665 %	1.6628 %	1.7209 %	1.6259 %	1.6047 %
	W	Extracellular structures	0.0697 %	0.0827 %	0.0186 %	0.0576 %	0.0203 %	0.0231 %	0.0823 %	0.0400 %	0.0202 %	0.1078 %	0.0196 %	0.0193 %	0.0189 %	0.0401 %	0.0406 %
	Z	Cytoskeleton	-	-	0.0186 %	0.0384 %	-	-	0.0206 %	0.0400 %	0.0202 %	0.0216 %	0.0392 %	-	-	0.0401 %	-

Table S2. tRNA of the *Planktothrix* genomes

tRNA	Ala	Arg	Asn	Asp	Cys	Gln	Glu	Gly	His	Ile	Leu	Lys	Met	Phe	Pro	Pseudo tRNA	Ser	Thr	Trp	Tyr	Val	Total
P. serta PCC 8927	4	3	1	1	1	1	1	3	1	2	4	1	3	1	3	-	4	3	1	1	3	42
P. tepida PCC 9214	5	4	1	1	1	2	1	3	1	2	5	2	3	1	3	1	4	3	1	1	3	48
P. paucivesiculata PCC 9631	5	3	1	2	1	2	2	3	1	3	4	2	3	1	3	-	4	3	1	1	3	48
Planktothrix sp. PCC 11201	5	5	1	1	1	1	1	3	1	3	4	1	3	1	3	1	5	3	1	1	3	48
P. agardhii PCC 7805	3	3	1	1	1	1	1	3	1	1	4	1	3	1	3	-	4	3	1	1	3	40
P. rubescens PCC 7821	3	3	1	1	1	1	1	3	1	1	4	1	3	1	3	-	4	3	1	1	3	40
P. agardhii NIVA-CYA 15	2	3	1	1	1	1	1	3	1	-	4	1	3	1	3	-	4	3	1	1	3	38
P. agardhii NIVA-CYA 34	2	3	1	1	1	1	1	3	1	-	4	1	3	1	3	-	4	3	1	1	3	38
P. agardhii NIVA-CYA 56-3	2	3	1	1	1	1	1	3	1	-	4	1	3	1	3	-	4	3	1	1	3	38
P. agardhii NIVA-CYA 126-8	3	3	1	1	1	1	1	3	1	1	4	1	3	1	3	-	4	3	1	1	3	40
P. mougeotii NIVA-CYA 405	2	3	1	1	1	1	1	3	1	-	4	1	3	1	3	-	4	3	1	1	3	38
P. prolifica NIVA-CYA 98	2	3	1	1	1	1	1	3	1	-	4	1	3	1	3	-	4	3	1	1	3	38
P. prolifica NIVA-CYA 406	2	3	1	1	1	1	1	3	1	-	4	1	3	1	3	-	4	3	1	1	3	38
P. prolifica NIVA-CYA 540	2	3	1	1	1	1	1	3	1	-	4	1	3	1	3	-	4	3	1	1	3	38
P. rubescens NIVA-CYA 407	2	3	1	1	1	1	1	3	1	-	4	1	3	1	3	-	4	3	1	1	3	38

References

1 Aury, J. M. *et al.* High quality draft sequences for prokaryotic genomes using a mix of new sequencing technologies. *BMC Genomics* **9**, 603, doi:10.1186/1471-2164-9-603 (2008).

2 Vallenet, D. *et al.* MicroScope--an integrated microbial resource for the curation and comparative analysis of genomic and metabolic data. *Nucleic Acids Res* **41**, D636-647, doi:10.1093/nar/gks1194 (2013).

3 Calteau, A. *et al.* Phylum-wide comparative genomics unravel the diversity of secondary metabolism in Cyanobacteria. *BMC Genomics* **15**, 977, doi:10.1186/1471-2164-15-977 (2014).

4 Achaz, G., Boyer, F., Rocha, E. P., Viari, A. & Coissac, E. Repseek, a tool to retrieve approximate repeats from large DNA sequences. *Bioinformatics* **23**, 119-121, doi:10.1093/bioinformatics/btl519 (2007).

5 Tatusov, R., Koonin, E. & Lipman, D. A genomic perspective on protein families. *Science* **278**, 631-637 (1997).

6 Blin, K. *et al.* antiSMASH 2.0--a versatile platform for genome mining of secondary metabolite producers. *Nucleic Acids Res* **41**, W204-212, doi:10.1093/nar/gkt449 (2013).

7 Bachmann, B. & Ravel, J. Chapter 8. Methods for in silico prediction of microbial secondary metabolic pathways from DNA sequence data. *Methods in Enzymology* **458**, 181-217 (2009).

8 Waack, S. *et al.* Score-based prediction of genomic islands in prokaryotic genomes using hidden Markov models. *BMC Bioinformatics* **7**, 142, doi:10.1186/1471-2105-7-142 (2006).

9 Vernikos, G. S. & Parkhill, J. Interpolated variable order motifs for identification of horizontally acquired DNA: revisiting the Salmonella pathogenicity islands. *Bioinformatics* **22**, 2196-2203, doi:10.1093/bioinformatics/btl369 (2006).
